# Feasibility of High-Frequency Ultrasound and Magnetic Resonance Imaging to Assess the In Ovo Development of Chicken Embryos

**DOI:** 10.3390/jimaging12050217

**Published:** 2026-05-20

**Authors:** Ylenia Ferrara, Cristina Terlizzi, Annachiara Sarnella, Luca Licenziato, Serena Monti, Marcello Mancini

**Affiliations:** Institute of Biostructures and Bioimaging, National Research Council, 80145 Naples, Italy; ylenia.ferrara@ibb.cnr.it (Y.F.); cristina.terlizzi@ibb.cnr.it (C.T.); luca.licenziato@ibb.cnr.it (L.L.); marcello.mancini@ibb.cnr.it (M.M.)

**Keywords:** preclinical research, imaging, chicken embryo, HFUS, MRI

## Abstract

Preclinical multimodal imaging is widely applied in small animal models for longitudinal studies of human diseases. Beyond murine systems, cost-effective and ethically sustainable models such as the chicken embryo and its chorioallantoic membrane are gaining increasing interest in accordance with the 3Rs principles. This study evaluated the feasibility of using both high-frequency ultrasound and magnetic resonance imaging for the non-invasive longitudinal monitoring of chicken embryo development in ovo. Fifty fertilized eggs were incubated under controlled conditions and examined up to embryonic day 14. High-frequency ultrasound (15–71 MHz) enabled real-time imaging and quantitative assessment of superficial structures, including cranial biometry and limb growth, while magnetic resonance imaging (7T) provided high-resolution three-dimensional visualization of internal organs and extraembryonic compartments. Together, these modalities allowed the progressive identification of key anatomical structures from ED5 onward, with HFUS enabling earlier linear measurements and MRI facilitating detailed anatomical and volumetric evaluation. The integration of these techniques allowed the generation of a developmental imaging timeline and quantitative reference dataset of normal embryogenesis. This multimodal approach represents a promising strategy for in vivo developmental studies, offering a robust baseline to characterize structural alterations induced by experimental conditions. Moreover, the use of the chicken embryo model provides significant ethical and economic advantages, supporting its application in preclinical research and imaging-based studies.

## 1. Introduction

The chicken embryo (CE) can be used in biomedical research for a variety of applications (evaluation of angiogenesis, tumor growth and response to therapy) due to its economic advantages, ease of access and ethical acceptability. Unlike other animal models, CEs require minimal housing—small incubators suffice—and physical access is straightforward since the only barrier is the eggshell, facilitating experimental manipulation and imaging [1,2,3,4,5]. The CE develops outside the maternal body, providing a powerful platform to study embryonic growth and development under physiological conditions free from maternal influences and external stressors. This accessibility enables continuous, noninvasive observation, as well as experimental manipulation throughout development. In addition, the chicken chorioallantoic membrane (CAM) model represents a robust and cost-effective system for investigating vascular processes, as it develops a functional circulatory network within three days of fertilization, can be cultured ex ovo, and supports the study of tumor growth, drug delivery, and the effects of anti-angiogenic agents.

For the optimal use of the CE model, it is essential to first characterize normal organ development and to define similarities and differences with other animal models, taking advantage of the possibility of performing repeated analyses over time without perturbing normal development [1,2,3,4,5].

Non-invasive imaging techniques have significantly advanced the study of CE development by allowing longitudinal, real-time morphological and functional analyses.

High-frequency ultrasound (HFUS), operating between 20 and 70 MHz with spatial resolution ranging from 30 to 100 µm, is a pivotal tool in this context. It enables a detailed visualization of critical developmental structures, including the neural tube, brain vesicles, heart, vasculature, limb buds, and extraembryonic tissues such as the yolk sac and allantois. HFUS also supports dynamic functional assessments, including heartbeat monitoring and blood flow analysis [1]. Its advantages include accessibility, cost-effectiveness, and the absence of ionizing radiation. Though HFUS’s penetration depth is limited compared to lower-frequency ultrasound, this limitation is offset by its superior resolution for superficial embryonic structures [6,7,8]. For instance, Alser et al. performed right atrial ligation on CEs at embryonic day 4 (ED4) to study the effect of flow perturbations on cardiogenesis. The authors used HFUS and echocardiography to provide quantitative, real-time measurements of flow velocity and integrated structural and functional cardiac data, demonstrating that HFUS is an ideal modality for analyzing hemodynamic perturbations in embryonic manipulation studies [9]. Tsimpaki et al. used HFUS as a monitoring tool to assess the growth of implanted tumors derived from patients and the presence and density of vessels within the tumor [10]. Furthermore, doppler ultrasound studies in the CEs have demonstrated the capability of ultrasound imaging to assess early cardiovascular development and vascular dynamics, supporting its application in angiogenesis-related investigations [11]. These application-driven studies support the capability of HFUS to capture both structural and functional dynamic changes. Compared to previous studies focused on specific stages or targeted applications, the present work provides a systematic longitudinal assessment across multiple embryonic days, enabling a more comprehensive characterization of physiological development.

Magnetic resonance imaging (MRI), in contrast, provides high-resolution imaging with excellent soft tissue contrast. Utilizing preclinical systems ranging from 7 T up to 18 T, MRI can capture detailed and even three-dimensional morphological data of both embryonic and extraembryonic tissues throughout incubation, from ED1 to hatching. MRI acquisition times are quite long, and the images can be affected by motion artifacts; consequently, the acquisition protocol should take this aspect into account to obtain good-quality images. However, MRI is able to provide whole-egg images, offering not only morphological measurement, but also functional and microstructural information [12,13,14,15]. Streckenbach et al. used MRI to quantify the volumes of the yolk/yolk sac, amniotic fluid, and CE in ovo [16], whereas Buschmann et al. characterized different tumors, by engrafting colon and adenocarcinoma cell lines in living CEs, in terms of their reaction to gas challenges. They used T1 and T2* mapping to enable differential characterization of tumor grafts with respect to their vascular and oxygenation status [17]. Chen et al. used MRI to monitor in ovo the morphological evolution of the CE, allantois and CAM longitudinally for all embryonic stages. Whole CE and allantois were segmented semi-automatically based on intensity-based threshold and region growth algorithms. 3D volumes of the segmented structures were quantified and confirmed by histological analysis (one for each ED) [14]. Considering the differences and complementarity of these two modalities, the purpose of this study was to evaluate the feasibility of HFUS and MRI for the non-invasive monitoring of CE development. A daily imaging protocol was implemented using both approaches, enabling the extraction of multiple anatomical and quantitative parameters from the acquired images. These measurements provided objective metrics for evaluating the capabilities and complementarity of each imaging modality. Furthermore, the systematic collection of imaging data allowed the creation of an image-based atlas of physiological CE development, which may serve as a reference framework for the identification of structural abnormalities affecting embryonic growth and organ function in future studies. From a practical and economic perspective, this multimodal approach provides a repeated, non-destructive assessment of the same embryo over time, reducing the number of animals required while improving experimental efficiency, longitudinal monitoring, and reproducibility across developmental stages.

## 2. Materials and Methods

### 2.1. Fertilized Eggs

A total of 50 fertilized Isa Brown eggs (Società Agricola Vinifera s.s., Forlì, Italy). Day 0 of embryonic development began when the eggs were placed in a horizontal position in an incubator with a relative humidity of 47 ± 2% at a temperature of 37.7 °C ± 0.2 °C (F.I.E.M. S.r.l., Como, Italy), inside which the eggs were automatically rotated (by 180°). Preliminary HFUS imaging, from ED1, was used to identify the earliest stage of anatomical visibility. Once the first structures were detectable, the imaging protocol started up to day 14 of development on n = 5 eggs (n = 3 for HFUS and n = 2 for MRI analysis) for each day of incubation, after checking CE viability by candling. At the end of each imaging session, the embryos were euthanized in accordance with applicable ethical guidelines. In line with the European Directive 2010/63/EU, avian embryos are not classified as protected animals until the last third of their development; therefore, procedures performed up to ED14 did not require formal approval from an institutional animal ethics committee.

### 2.2. Non-Invasive High-Frequency Ultrasound

**Figure 1:**
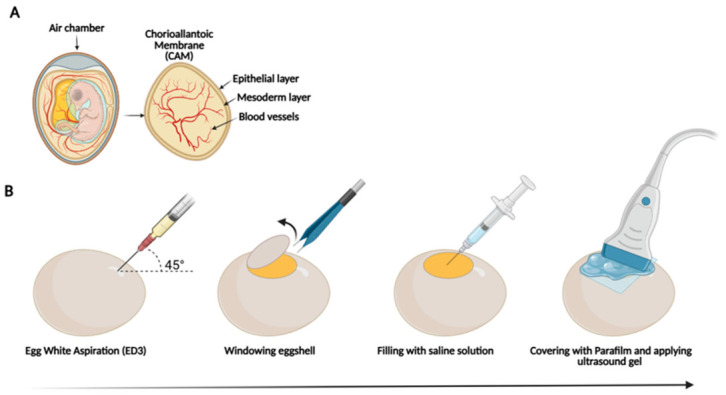
Schematic representation of CE preparations for HFUS procedure. (**A**) Graphical illustration of the CE. (**B**) Schematic representation of the steps for preparing the CE for HFUS analysis (modified from [1] and created with Biorender).

An HFUS system (VEVO F2, FUJIFILM VisualSonics, Inc., Toronto, ON, Canada) was used with ultra-high-frequency transducers UHF71x (71–30 MHz), UHF57x (57–25 MHz) and UHF29X (29–15 MHz) (FUJIFILM VisualSonics) to assess the morphology and structural development of CEs from ED5 to ED14. For the analysis, on embryonic day 3, after locating the air chamber using the candling technique, a small hole was made at the opposite pole of the egg to allow removal of approximately 4 mL of albumen using a 10 mL syringe. This is an optional preparatory step commonly performed to slightly reduce the internal volume and pressure of the egg, causing a slight detachment of the CAM and the developing embryo from the inner shell membrane. This facilitates a safer shell window, improves visualization during the subsequent ultrasound, and minimizes the risk of accidental rupture of the CAM or bleeding during shell opening. The removed albumen was discarded, as its removal was performed only to create additional space inside the egg and was not used for further analysis. The HFUS evaluation was conducted in ovo at room temperature without anesthesia. Before imaging, the position of the CE inside the egg was evaluated under light to angle the transducer according to the possible location of the embryo [18]. The eggshell was carefully opened with fine forceps to the maximum possible extension without injuring the CAM, thereby minimizing the risk of injury to the embryo, and the egg was filled with physiological solution. Specifically, the opening was tightly covered with parafilm, and ultrasound gel (GIMA) was placed over it to allow visualization of structures without directly affecting the embryo (Figure 1). Brightness (B-) mode was used to identify the acquisition plane and to track the growth of anatomical structures such as brain, cardiac structures, abdominal organs and bones using the following setting: Gain 0–3 dB; Dynamic Range 54.17–57.9 dB; Brightness 50; Contrast 50. Images were acquired at least three times for each structure of every embryo in the paracoronal and longitudinal planes; the total scanning time was about 20 min.

### 2.3. Magnetic Resonance Imaging

MRI scans were performed on an MRS*DRYMAG 7.0T, a cryogen-free MR scanner based on the proprietary Dry Magnet technology from MR Solutions that does not require liquid helium for the cooling, equipped with a transmit/receive volume coil for rat body imaging. Before the acquisition, the embryos were placed in a fridge at 4 °C for 60 min to minimize motion artifacts. Fast positioning imaging was performed using a T2-weighted fast spin echo (FSE) sequence to obtain three orthogonal planes. Next, two high-resolution whole-body FSEs were acquired according to sagittal and coronal brain plane orientation (TR/TE 3789/25 ms, FOV 4.5 cm, matrix 256 × 238, ETL 7, NEX 3, slice thickness 0.5 mm, no gap, No. of slices 40, approximately 8 min duration).

### 2.4. Image Processing and Statistical Analysis

All acquired images were stored in the DICOM format for offline data analysis using Vevo LAB software (Version 5.7.1, Fujifilm Visualsonics Inc., Canada) and 3D Slicer image computing platform (Version 5.8.1, Slicer Brigham and Women’s Hospital, Harvard University, NIH) for HFUS and MRI, respectively. Anatomical identification and measurements were performed with reference to the *Atlas of Chick Development* (Third Edition) [19]. Mesencephalic and diencephalic cavities, beak, third toe, heart, liver, biparietal distance (BPD), biorbital distance (BOD), and abdominal circumference were measured three times per embryo. Values are reported as mean ± standard deviation (SD) of all measurements.

### 2.5. GenAI

Generative artificial intelligence (AI; ChatGPT version GPT-5.5 di OpenAI) was used to assist with language refinement and stylistic revision of selected sections of the manuscript, such as figure legends. No AI tools were used for data generation, analysis, figure preparation, or scientific interpretation. All content was critically reviewed and approved by the authors, who take full responsibility for the manuscript.

## 3. Results

**Figure 2:**
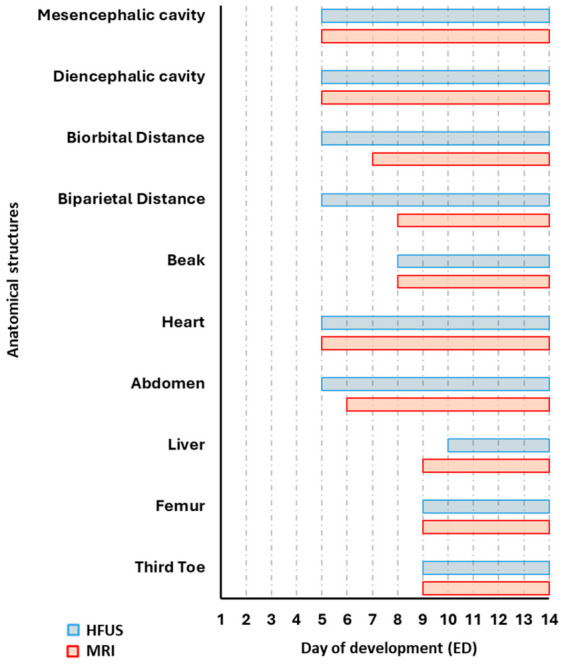
Gantt chart of anatomical structure visibility assessed by HFUS and MRI.

Preliminary HFUS imaging revealed that, up to ED4, no distinct structures were detectable (Figure S1). From ED5 onwards, distinct anatomical features progressively emerged, enabling systematic imaging-based analysis and measurements. Consequently, development day 5 was selected as the starting point for both HFUS and MRI. At this stage, the CAM became increasingly visible due to its rapid vascularization and expansion, providing a well-defined and accessible structure for imaging. From ED5 to ED14, both HFUS and MRI allowed for the progressive characterization of organogenesis, vascular development and structural maturation. The chart in Figure 2 shows the embryonic days at which specific anatomical structures first became detectable and could be clearly identified using these imaging modalities. This timeline provides a clear overview of the sequential onset of various structures, reflecting the dynamic nature of embryogenesis and the evolving capability of these two imaging approaches to resolve anatomical detail as development advances. Visualization helps to delineate the developmental window during which each structure can be reliably identified, thus serving as a practical reference for future longitudinal studies involving the in ovo imaging of CEs.

### 3.1. HFUS

**Figure 3:**
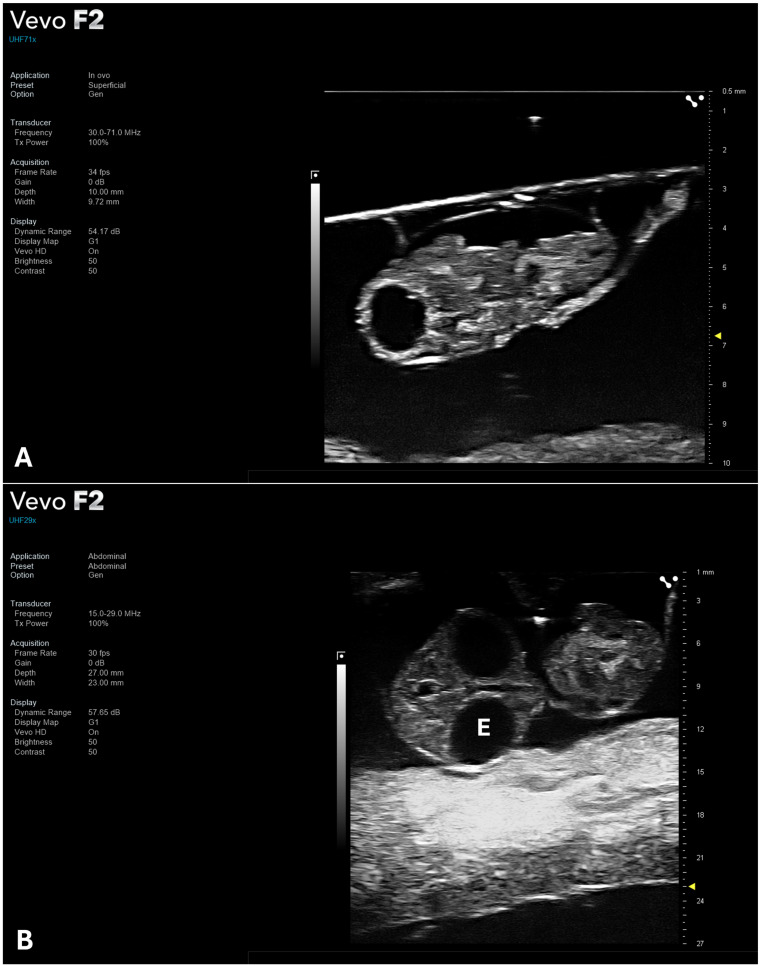
Representative frames of CE full-length at ED5 (**A**) and ED8 (**B**). Acquired using UHF71X probe (FUJIFILM VisualSonics) in paracoronal plane. Abbreviation: E, eye.

The HFUS evaluation was conducted on 30 eggs (n = 3 eggs per ED). Accurate measurements of the full embryonic length were feasible only between ED5 and ED9, primarily due to the positioning of the CE within the confines of the eggshell during development (Figure 3). As the embryo progresses, it adopts a specific orientation, with the head tucked beneath the right wing and directed toward the blunt, air-cell-containing end of the egg. This arrangement, commonly referred to as the “pipping” position, is essential for the hatching process, as it enables the embryo to exert pressure on the air cell using its beak to initiate external pipping [20]. However, this natural positioning restricts the ability to obtain linear biometric measurements, particularly in advanced stages of development (Figure 3).

**Figure 4:**
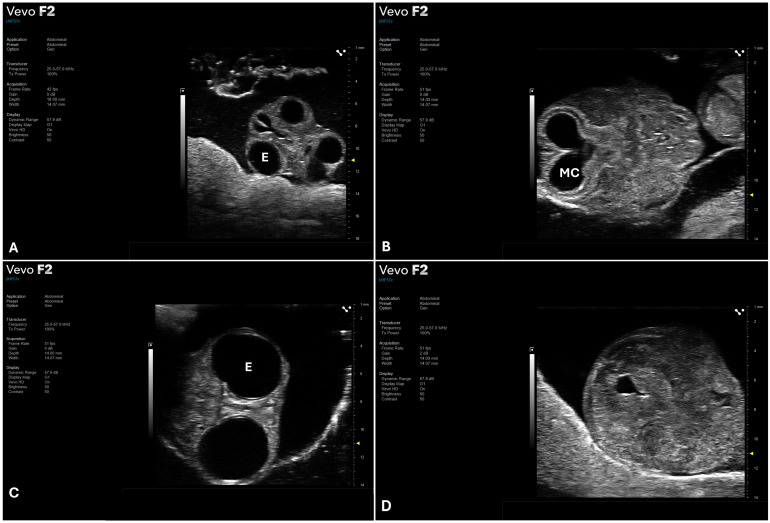
Representative frames of CE entire cranial structure at ED6 (**A**), mesencephalic cavity at ED8 (**B**). Orbits (**C**) and parietal region (**D**). Acquired using UHF57X probe (FUJIFILM VisualSonics) in paracoronal plane. Abbreviations: E, eye; MC, mesencephalic cavity.

From ED5 onward, it was possible to identify developing heart and major brain structures such as the mesencephalic cavity and the diencephalic cavity [21]. At this stage, the CAM also became visible as a peripheral outline surrounding the embryo, reflecting its progressive expansion and vascularization, providing an additional anatomical landmark for imaging. Although mature brain structures derived from mesencephalon and diencephalon begin to differentiate by ED8, detailed visualization of their internal subdivisions remains challenging due to the limited contrast resolution of HFUS and variability in embryo positioning within the egg. At this stage, cranial biometry was achievable through the measurements of BOD, determined by the outer margins of the orbital bones, and BPD, assessed at the level of the optic lobes in paracoronal plane (Figure 4 and Figure 6).

**Figure 5:**
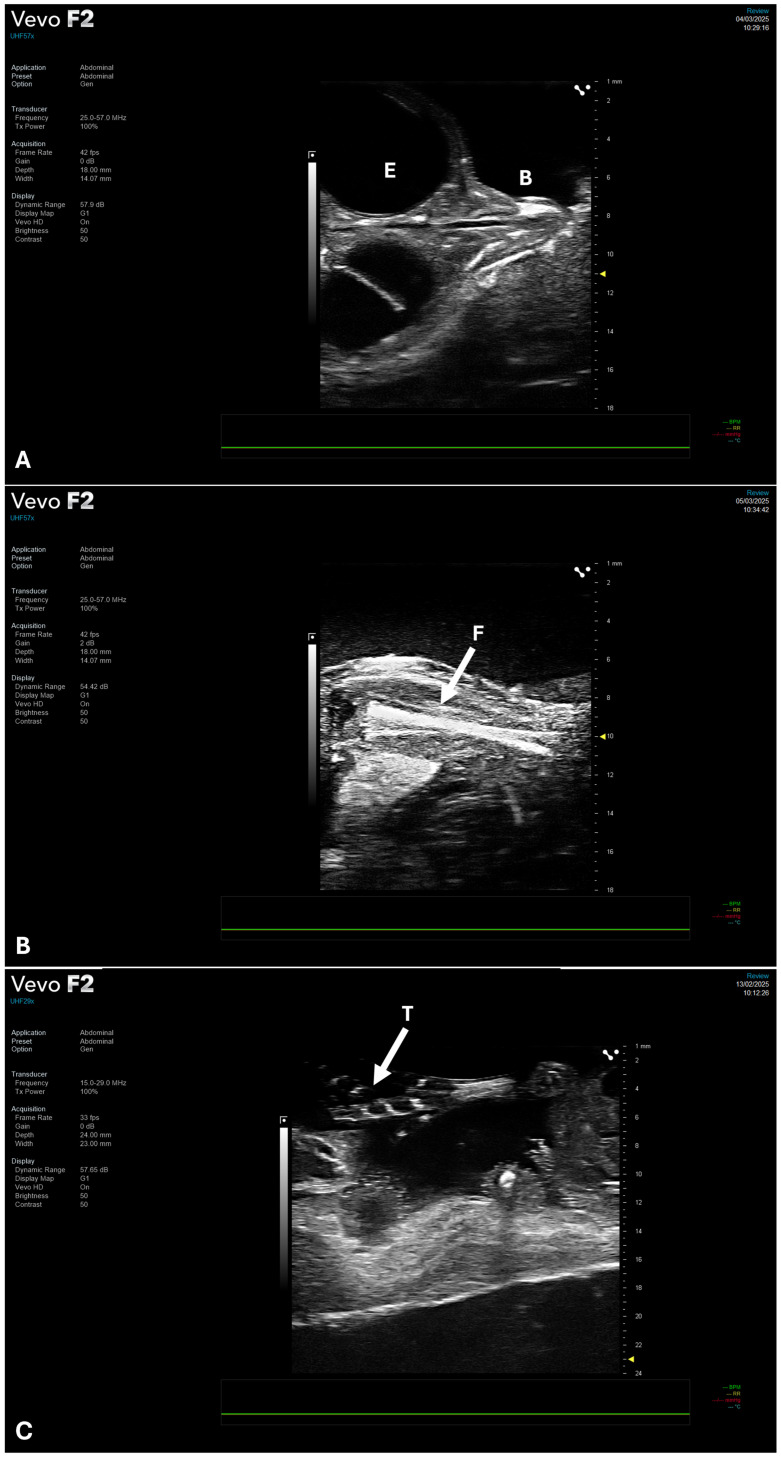
Representative frames of CE beak (**A**), femur (**B**) and third toe (**C**). Acquired using UHF57X or UHF29X probes (FUJIFILM VisualSonics) in paracoronal plane for the beak and longitudinal plane for femur and third toe. Abbreviations: E, eye; B, beak; F, femur; T, third toe.

**Figure 6:**
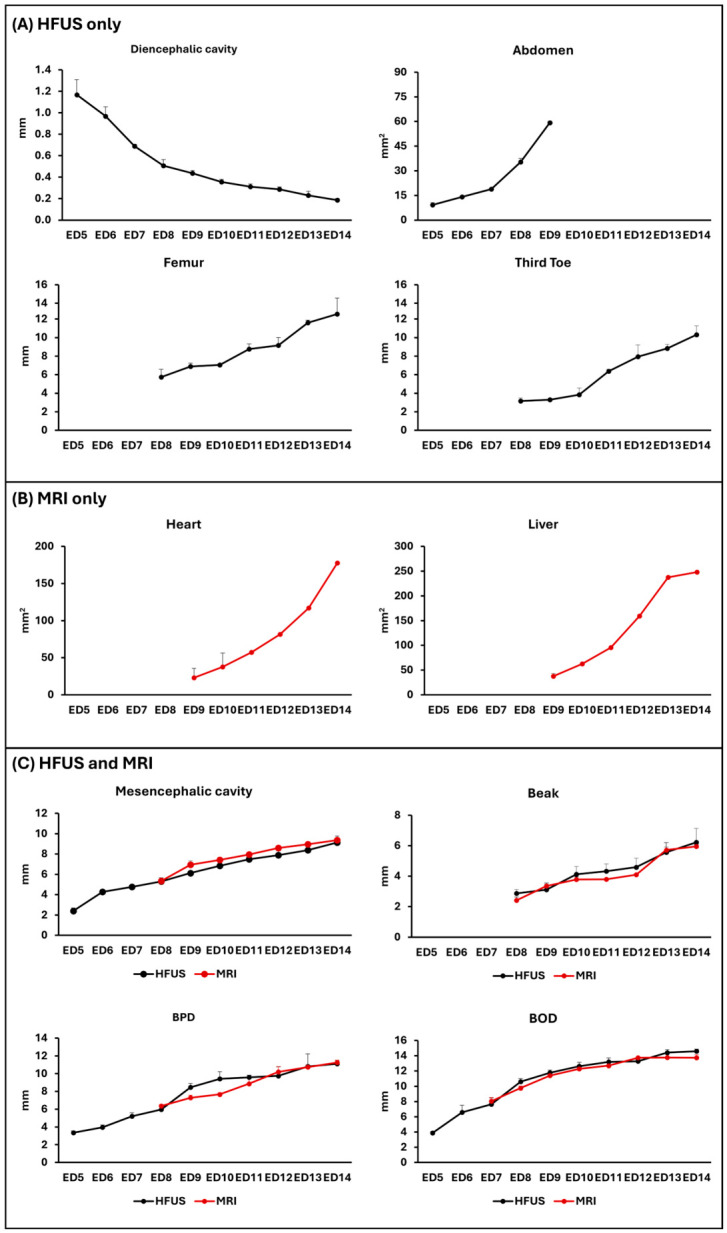
Morphometric growth curves during embryonic development. (**A**) Daily HFUS growth measurements of diencephalic cavity, abdomen, femur and third toe, expressed in mm/day. (**B**) Daily MRI volume measurements of the heart and liver, expressed in mm/day. (**C**) Overlapping growth curves of mesencephalic cavity, beak, biparietal distance (BPD) and biorbital distance (BOD) measurable by both HFUS and MRI during CE development, expressed in mm/day.

By ED8, distinct anatomical features such as the beak and femur became visible and were subsequently monitored over time for growth assessment (Figure 5). Although abdominal organs remained poorly distinguishable with HFUS at this stage, ED9 marked the final day on which it was possible to reliably measure the abdominal circumference using the available ultrasound transducers (UHF71x, UHF57x and UHF29X). Between ED10 and ED14, further anatomical maturation made the liver and pectoral muscles visible (Figure S2). The third toe also becomes clearly identifiable in this epoch, and its length, measured from the metatarsophalangeal joint to the tip of the claw, was measured, offering an additional developmental parameter for longitudinal assessment (Figure 5 and Figure 6).

Figure 6A,C show the daily growth trends of different anatomical structures evaluated by HFUS during CE development (diencephalic cavity, abdomen, femur, third toe, mesencephalic cavity, beak, BPD and BOD). The graphs highlight their progressive growth and the different growth dynamics observed across the examined developmental period.

### 3.2. MRI

The MRI evaluation was successfully conducted on 20 CEs (n = 2 per ED). In this study, MRI was employed to observe the progressive development of CEs from ED5 to ED14. MRI enables the precise visualization and evaluation of major developing organs, including the brain, heart, liver, and extraembryonic structures, without the limitations posed by acoustic window or embryo orientation. Figure 6B shows the longitudinal MRI-based volumetric measurements of the heart and liver, whereas Figure 6C reports daily measurements of the growth of key anatomical structures (mesencephalic cavity, beak, BPD and BOD) assessed by MRI. The graphs illustrate the progressive morphological changes and the increase in size of the structures analyzed throughout the entire developmental period.

**Figure 7:**
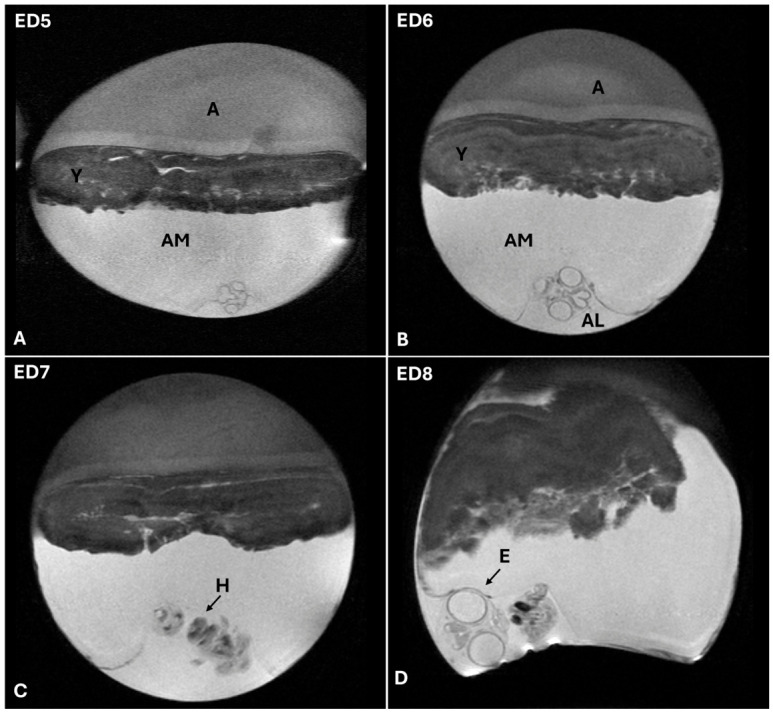
T2-weighted MRI images of CE between ED5 and ED8 showing clear differentiation of egg compartments (**A**,**B**) and progressive visualization of key embryonic structures, including heart (**C**) and eyes (**D**), in paracoronal planes. Abbreviations: AM, amniotic cavity; A, albumen; Y, yolk; AL, allantois; H, heart; E, eye.

Between embryonic ED5 and ED8, MRI allows clear differentiation of the egg’s internal compartments, such as the yolk sac, amniotic fluid, albumen, and CAM, due to the distinct signal intensities produced by the various fluid-filled and tissue-rich structures. During this ED window, several key embryonic features begin to emerge with increasing clarity. Eyes, developing brain regions, and cardiac structures become visible, allowing early assessment of the formation of the central nervous and cardiovascular systems. Although abdominal organs such as the liver and gastrointestinal tract begin to develop at this stage, their precise anatomical boundaries remain poorly defined because of ongoing organogenesis and limited contrast between surrounding tissues. However, early structural differentiation can be effectively monitored with MRI during this critical stage of chick embryogenesis, setting the stage for more detailed assessments in later days as organ systems mature (Figure 7).

**Figure 8:**
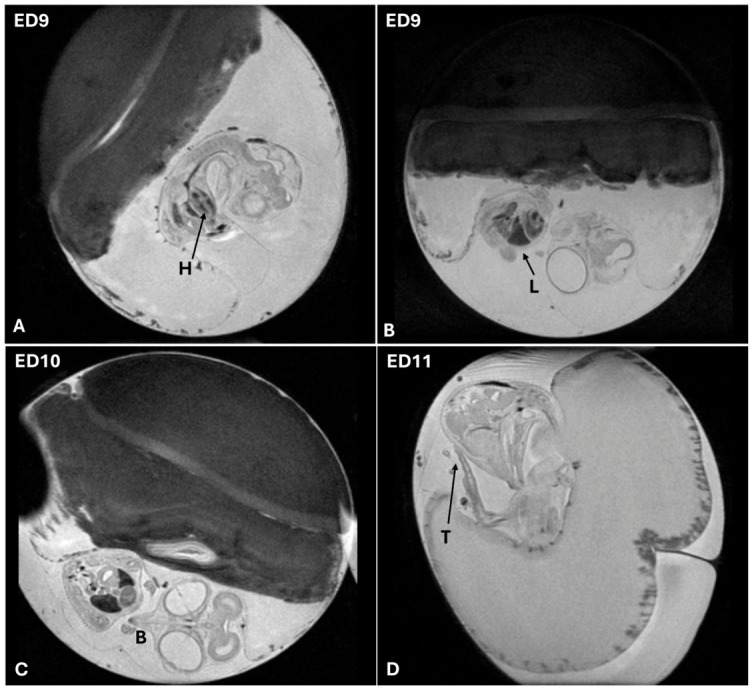
(**A**–**D**) T2-weighted MRI images of CE between ED9 and ED11 showing reduction in yolk sac and albumen volumes. Images acquired in sagittal and paracoronal planes. Increased anatomical detail is evident, including a more defined beak, visible liver within the thoracic cavity, femur growth, and measurable third toe. Abbreviations: H, heart; L, liver; B, beak; T, third toe.

From ED9 to ED12, there is an accelerated growth and morphological differentiation. This rapid development is accompanied by a marked reduction in the volumes of the yolk sac and albumen, which serve as the primary sources of nutrients and hydration. Their progressive depletion reflects the embryo’s increasing metabolic demands as organogenesis advances and physiological functions become more complex. Additional anatomical structures become detectable: the beak becomes more defined, indicating craniofacial development, while the liver begins to take shape and is distinguishable within the thoracic cavity. The femur also exhibits significant growth and differentiation, and the third toe can be measured and monitored as an external marker of limb development (Figure 8).

**Figure 9:**
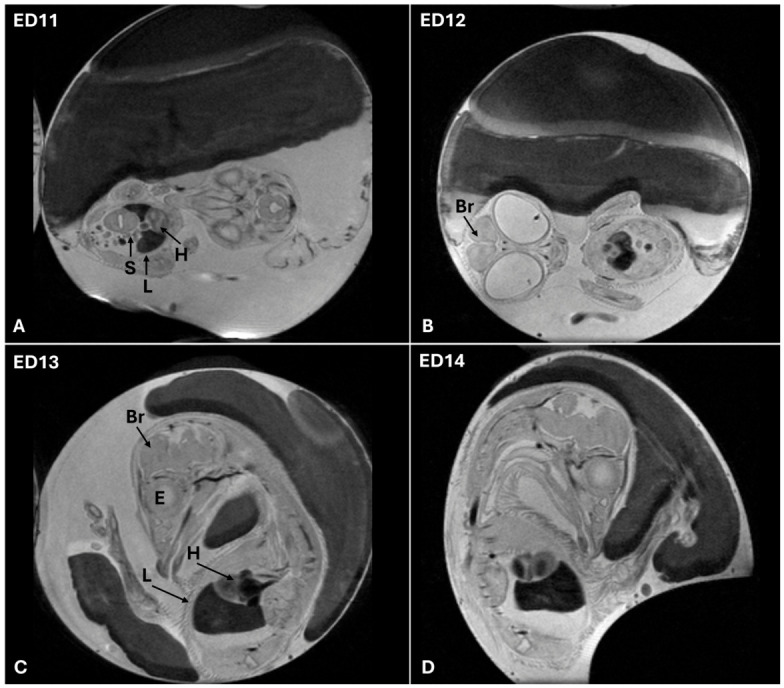
(**A**–**D**) T2-weighted MRI representative images of CE at ED11–ED14 showing advanced morphological maturation. Images were acquired in sagittal and paracoronal planes. Abbreviations: H, heart; L, liver; S, stomach; Br, brain; E, eye.

By ED13 and ED14, the CE reaches an advanced stage of development characterized by pronounced morphological and physiological maturation. At this stage, internal organs such as the heart, liver, and portions of the gastrointestinal tract become more clearly defined and occupy their respective anatomical positions within the thoracic and abdominal cavities. These structures exhibit increased tissue differentiation and organization, making them more readily identifiable through MRI. The beak becomes sharply contoured, and limb development progresses, with clear visibility of toes, claws, and joint articulations. In addition, the appearance of the outer coat, consisting of feather follicles and developing down feathers, marks a notable shift toward the embryo’s preparation for hatching. These cutaneous features, along with enhanced muscular definition, contribute to the overall somatic maturity of the embryo (Figure 9).

## 4. Discussion

HFUS and MRI are valuable tools for monitoring growth and evaluating the anatomical development of CEs. HFUS is particularly effective during the early stages of development, as it provides a broad overview of the embryo when internal organs are still immature and not yet distinguishable in detail. Despite this, several studies have shown that HFUS can already resolve key anatomical features at early developmental stages, including components of the developing cardiovascular system [8], enabling clear visualization of major chambers, such as the atria and ventricles, as well as the real-time evaluation of hemodynamics [22]. These findings highlight the utility of ultrasound as a powerful and noninvasive imaging modality for the real-time monitoring of both normal physiological functions and disease-related processes. Its ability to provide high-resolution imaging makes it a valuable tool in several research areas, including developmental biology, oncology, and cardiovascular studies. MRI, in contrast, complements HFUS by offering precise, high-resolution imaging throughout early development. MRI has emerged as a robust tool for studying CE development, offering excellent soft-tissue contrast, three-dimensional morphological information, and the advantage of avoiding ionizing radiation. Recent work has demonstrated that MRI enables comprehensive visualization of the entire embryo and the progressive formation of internal organs from their earliest appearance through late development [14]. Moreover, micro-MRI techniques have proven effective for the detailed anatomical characterization of avian embryos, including the central nervous system and visceral organs [3]. Unlike HFUS, MRI is not affected by embryo orientation or acoustic interference, making it a reliable modality for longitudinal anatomical assessment; its primary limitations are the need to minimize motion, often requiring immobilization, and longer acquisition times compared to HFUS.

In this study, we employed both HFUS and MRI to demonstrate the feasibility and effectiveness of these non-invasive imaging modalities for the assessment of CE in ovo development. Through the integration of these two complementary techniques, we were able to establish a comprehensive set of growth parameters and morphological reference data that characterize normal embryonic development. These parameters include both structural indicators obtained from longitudinal ultrasound imaging, as well as high-resolution anatomical data derived from MRI scans. The two modalities appear largely comparable in terms of the developmental stage at which organ differentiation can first be visualized (ED5) consistently, with previous reports showing early visualization of embryonic structures using super-resolution and contrast-enhanced ultrasound [23] and early MRI-based anatomical mapping of avian embryos [3]. However, HFUS demonstrates the ability to measure BPD and BOD two/three days earlier than MRI, likely because of its superior temporal resolution and sensitivity to superficial morphological changes. Both modalities provide equivalent capacity for visualizing major brain sub-divisions starting from ED5, in agreement with MRI-based longitudinal studies that have documented the early detection of telencephalic and cerebellar structures [12], thus highlighting their complementary roles in the longitudinal assessment of embryonic development. Quantitative measurements could only be obtained for a subset of the analyzed anatomical structures (mesencephalic cavity, beak, BOD and BPD) using both HFUS and MRI (Figure 6C). This limitation mainly reflects the intrinsic technical differences between the two imaging modalities, as well as the progressive anatomical complexity of the developing embryo. Furthermore, the differences in the anatomical structures measurable by HFUS and MRI were largely influenced by embryo orientation within the egg, which directly affected the feasibility of acquiring reliable and reproducible measurements with both modalities.

Our observations are also consistent with classical anatomical descriptions of chick embryogenesis, in which the progressive differentiation of cranial, visceral, and limb structures becomes increasingly evident between ED5 and ED14 [19]. In particular, the temporal appearance of the beak, developing limbs, and brain subdivisions observed with HFUS and MRI closely reflects previously described embryological staging patterns. Compared with conventional histological approaches, the multimodal imaging strategy adopted in this study provides the additional advantage of enabling the longitudinal and non-destructive assessment of the same embryo over time, thereby reducing inter-sample variability and preserving the physiological developmental context.

While HFUS enables accurate linear measurements of superficial and dynamically accessible structures, MRI provides a more comprehensive anatomical overview and facilitates the assessment of deeper structures. Notably, MRI also enables volumetric evaluation, an aspect that is more challenging to achieve with ultrasound due to acoustic constraints and embryo positioning. Recent ultra-high-field MRI studies have demonstrated the feasibility of detailed volumetric quantification of embryonic compartments and tissues throughout development [16], further highlighting the strengths of MRI for three-dimensional anatomical assessment. These differences highlight the complementary roles of the two techniques in imaging embryonic development. Our findings align with previous studies demonstrating the utility of ultrasound for high-resolution vascular and microstructural imaging in CEs [23] and the value of MRI for detailed anatomical and volumetric characterization across multiple developmental stages [3,12,16]. However, this study expands the current knowledge by systematically integrating these two imaging modalities within the same experimental framework, enabling a direct comparison of their performance and complementary roles. The integration of HFUS and MRI therefore represents a significant advantage, as the strengths of one modality compensate for the limitations of the other, enabling a more complete morphological and quantitative characterization of CE development. To our knowledge, this is one of the first studies to combine HFUS and MRI for longitudinal in ovo imaging of CE development, providing both morphometric and volumetric reference data within a unified approach.

### 4.1. Limitations

The combined HFUS–MRI dataset provides a robust baseline for evaluating the progression of embryogenesis under standard conditions. Despite the strengths of our study, several limitations should be acknowledged. A key consideration is our decision to limit analysis between ED5 and ED14. Before ED5, indeed, the small size and minimal tissue differentiation of the embryo fall below the minimum spatial and contrast resolution of both HFUS and MRI under the experimental conditions applied. In addition, in this phase, embryonic structures are not yet well delineated, and the amniotic cavity appears brighter than the surrounding albumen and yolk in MRI, suggesting a higher water content in the amniotic fluid. Most importantly, the ED14 upper limit was chosen in accordance with current ethical guidelines, as CEs up to this stage are generally not considered capable of perceiving pain. In line with this, the European Directive 2010/63/EU excludes avian embryos in the early stages of development from its regulatory scope. According to the directive, animal welfare legislation does not apply to avian embryos until they reach a stage of development at which they are capable of independent life and the perception of pain, typically identified as the final third of the incubation period [24,25,26]. By restricting our analysis to ED14, we ensured compliance with these ethical standards while minimizing potential distress to the embryos. From a technical standpoint, HFUS imaging presented challenges primarily related to embryo accessibility and acoustic limitations. To consistently achieve optimal signal quality, precise shell fenestration was required to access the embryo without compromising its viability, as the embryo’s position within the egg significantly affected image acquisition [8]. In addition to these practical difficulties, there are the intrinsic physical limitations of HFUS: increasing the frequency improves spatial resolution but significantly reduces penetration depth due to frequency-dependent acoustic attenuation [27]. Consequently, deeper or more advanced anatomical structures have become progressively more difficult to visualize. Additionally, the resolution and penetration limits of our available ultrasound transducers reduced the detectability of several structures at later developmental stages, emphasizing the need for alternative ones. Together, these factors highlight the necessity for next-generation HFUS technologies with improved penetration depth, adaptive beamforming, or contrast-enhanced approaches to achieve a more consistent visualization of late-stage embryonic anatomy. MRI, while offering superior soft tissue contrast and volumetric capabilities, also introduced methodological considerations. Cooling the eggs effectively minimized motion artifacts during scanning, but this intervention may have caused slight changes in the volumes of the compartments inside the egg or in tissue hydration. However, this did not appear to adversely affect overall embryonic development. Another limitation is the relatively small sample size, which is sufficient for a proof-of-concept study but may not fully capture the range of biological variability. Furthermore, we did not examine potential correlations between the dataset of growth measurements and egg size or weight, which could be an interesting area for future research given the morphometric diversity of chicken eggs [28].

### 4.2. Future Perspectives and Conclusions

The integration of HFUS and MRI opens up new possibilities for the longitudinal and non-invasive study of vertebrate embryogenesis, providing a powerful tool for investigating both normal development and pathological or experimentally induced disruptions. Future research could leverage this multimodal approach to characterize disease models, assess the impact of pharmacological or environmental exposures, and establish standardized imaging-based developmental biomarkers. In addition, although three-dimensional ultrasonography was beyond the scope of this preliminary feasibility study, its future implementation could further enhance HFUS capabilities by improving the spatial visualization and volumetric assessment of embryonic structures. The integration of 3D ultrasound approaches may therefore expand the applicability of HFUS for longitudinal developmental imaging and provide additional quantitative information complementary to MRI. Starting from this foundation, we also intend to expand the imaging platform by incorporating advanced optical imaging modalities that offer resolution from the cellular to the tissue level and are particularly well suited for visualizing vascular development, neural organization, and dynamic morphogenetic processes that remain difficult to capture with HFUS or MRI alone. Optical imaging would further enhance the analytical depth of the pipeline by enabling subcellular and molecular visualization, quantitative mapping of vascular and neural networks, high-speed imaging of rapid morphogenetic events, and ex vivo validation of HFUS and MRI results using clarified tissue sections. The integration of optical imaging with HFUS and MRI would thus create a truly multimodal and multiscale platform capable of capturing the morphology of the entire embryo, fine-scale tissue architecture, and dynamic developmental processes. In combination with advanced computational tools, including automated segmentation, machine learning-based phenotyping, and multimodal data fusion, this expanded framework could substantially increase the accuracy, productivity and translational relevance of embryonic imaging. In conclusion, HFUS and MRI represent complementary, non-invasive modalities that, together, provide a robust and ethically sustainable platform for studying CE development in vivo. Overall, our work supports the refinement of CE analysis and reinforces the ethical principles of the 3Rs (Replacement, Reduction, and Refinement), contributing to more precise and ethically aligned approaches for studying vertebrate development.

## Data Availability

The data supporting the findings of this study are available from the corresponding authors upon reasonable request via email.

## Supplementary Materials

The following supporting information can be downloaded at https://www.mdpi.com/article/10.3390/jimaging12050217/s1: Figure S1. Representative HFUS images of ED1–ED3 of CEs development; Figure S2. Visualization of heart, liver, pectoral muscles and abdomen by HFUS.
