# Supplementary figures and images for: Feasibility of High-Frequency Ultrasound and Magnetic Resonance Imaging to Assess the In Ovo Development of Chicken Embryos
Source: J Imaging. 2026 May 20;12(5):217. doi: 10.3390/jimaging12050217 (PMC13207719; doi:10.3390/jimaging12050217)

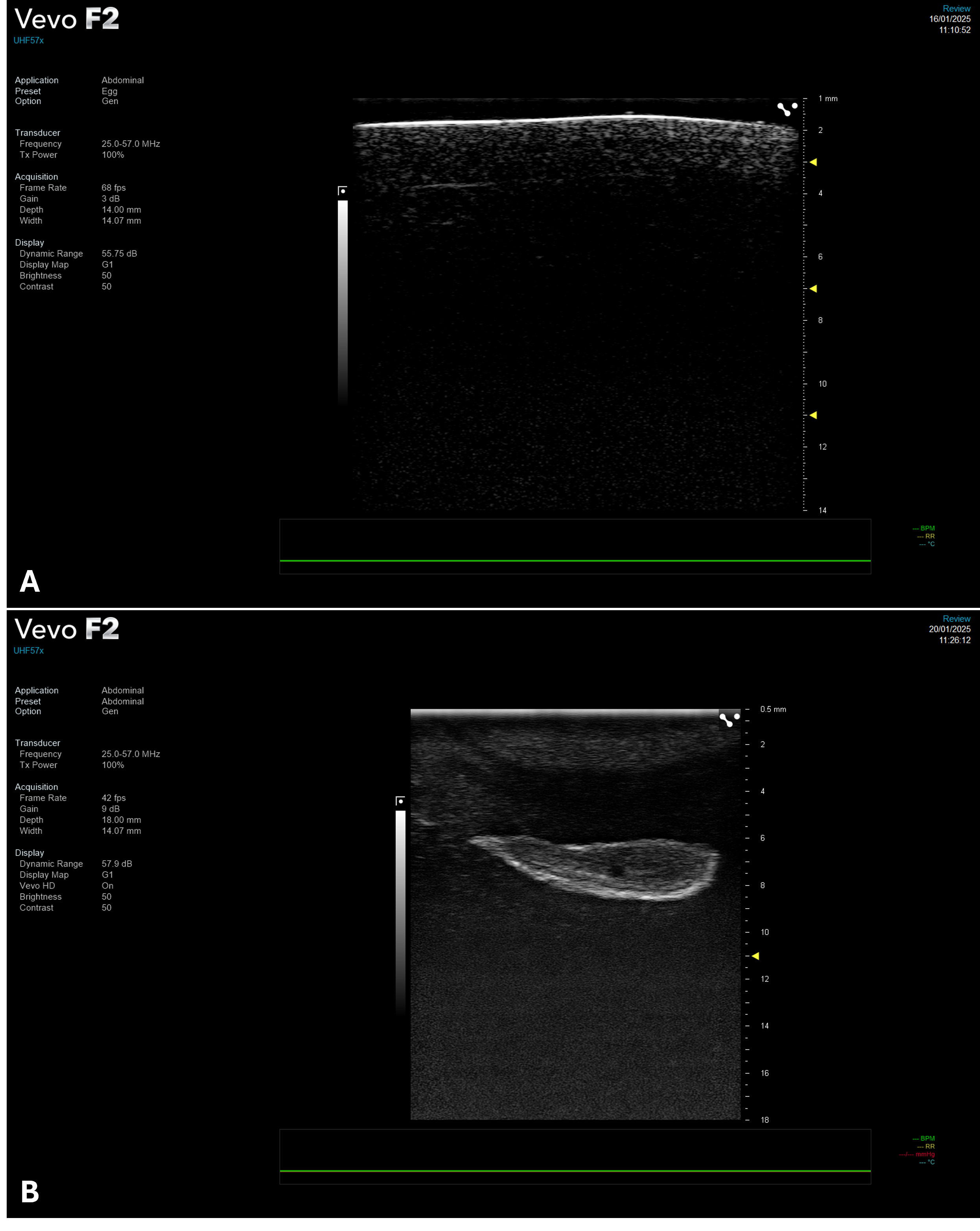

Supplement: Supplementary file 1 [file jimaging-12-00217-s001.zip › Figure S1_revised.png]

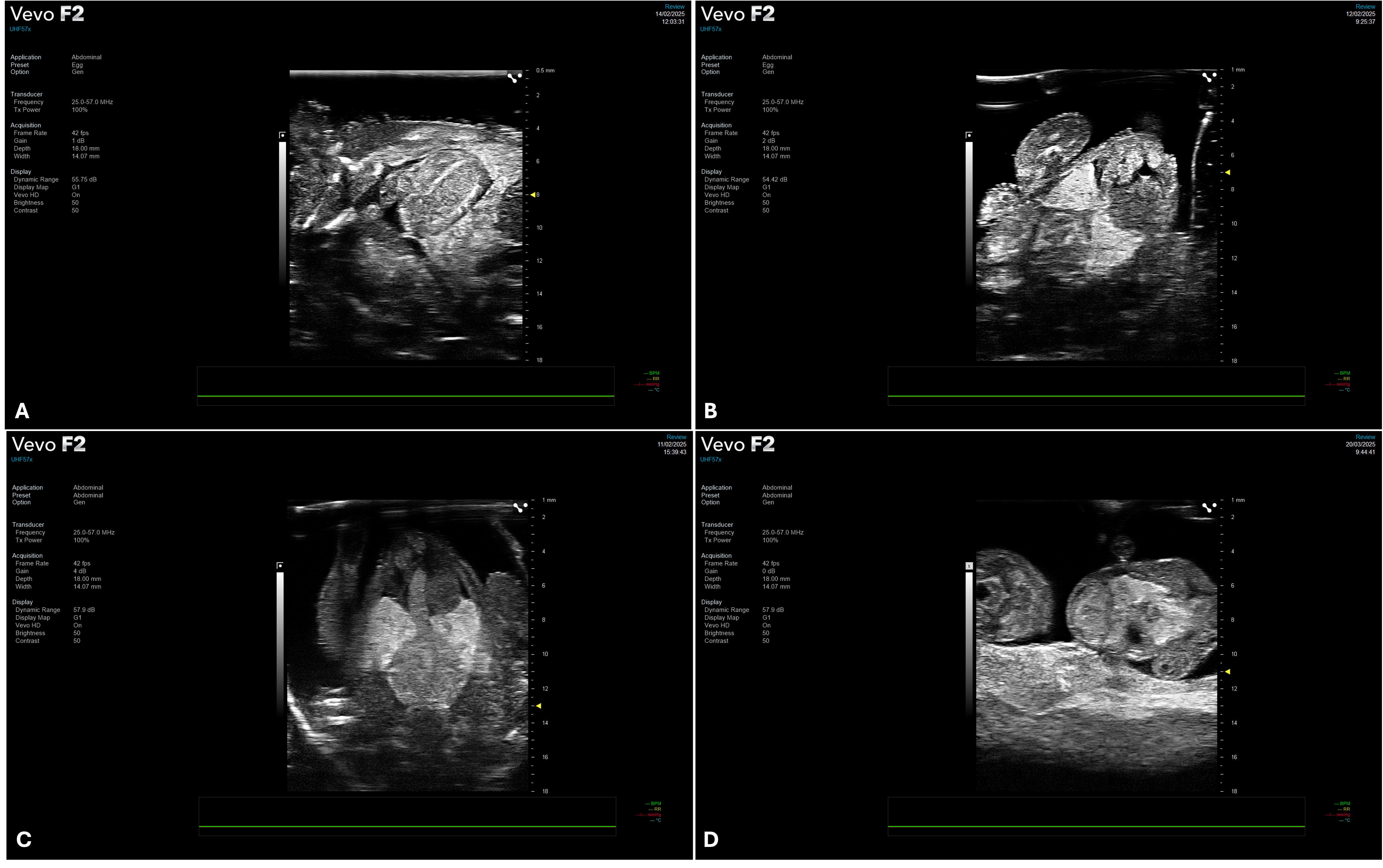

Supplement: Supplementary file 1 [file jimaging-12-00217-s001.zip › Figure S2_revised.png]
